# Recurrent Ischaemic Stroke Secondary to Cerebral Small Vessel Vasculopathy From Clonal B-Cell Lymphomatosis of Marginal Zone Origin

**DOI:** 10.1155/crnm/6620797

**Published:** 2024-11-23

**Authors:** Rebecca Hui Min Hoe, Zheyu Xu, Rajinder Singh

**Affiliations:** Department of Neurology, National Neuroscience Institute (Tan Tock Seng Hospital Campus), Singapore

**Keywords:** cerebral vasculopathy, clonal B-cell lymphomatosis of marginal zone origin, marginal zone lymphoma, recurrent ischaemic stroke

## Abstract

Marginal zone lymphoma (MZL) is an indolent lymphoma that rarely involves the central nervous system (CNS). Clonal B-cell lymphomatosis of marginal zone origin (CBL-MZ) is a premalignant condition referring to the presence of clonal B cells in the peripheral blood without evidence of organomegaly, lymphadenopathy or other features of established lymphoma, which may uncommonly progress to MZL, and as such does not require treatment beyond active surveillance. A 54-year-old male with previously diagnosed CBL-MZ presented with multiple recurrent subcortical ischaemic strokes. There was no evidence of progression to overt MZL or secondary transformation on repeated evaluation. His strokes proved refractory to antithrombotic therapy and anticoagulation. The absence of significant cardiovascular risk factors led to an extensive evaluation which excluded secondary causes such as cardioembolism, prothrombotic state or systemic vasculitis. Eventually, he was found to have lymphomatous involvement of the cerebrospinal fluid. The recurrent ischaemic strokes were attributed to a cerebral small vessel vasculopathy from neoplastic meningitis, which prompted the initiation of chemotherapy, leading to a remarkable cessation of stroke recurrence. This case highlights the importance of considering CNS involvement even in indolent or premalignant lymphomas when these patients present with “cryptogenic” recurrent strokes that appear refractory to standard secondary stroke prevention therapy. We also describe the approach to recurrent ischaemic stroke, the importance of imaging to determine the stroke mechanism, and the approach to small vessel cerebral arteriopathies.

## 1. Introduction

Marginal zone lymphoma (MZL), an indolent lymphoma, rarely involves the central nervous system (CNS). Clonal B-cell lymphomatosis of marginal zone origin (CBL-MZ) is a premalignant condition referring to the presence of clonal B cells in the peripheral blood without evidence of organomegaly, lymphadenopathy or other features of established lymphoma, which in 15%–20% of cases may progress to MZL, and as such does not require treatment beyond active surveillance [1]. We report a challenging case of CBL-MZ with CNS involvement in the form of cerebral small vessel vasculopathy and discuss the clinical approach to evaluation and treatment.

## 2. Case Report

**Figure 1:**
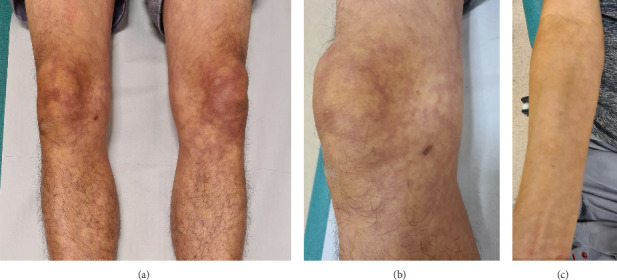
Series of images demonstrating (a): livedo reticularis on bilateral lower limbs, (b): close-up image of livedo reticularis on the right lower limb, and (c): livedo reticularis on the right upper limb.

A 54-year-old Chinese man was diagnosed with CBL-MZ following the appearance of livedo reticularis (Figure 1). Skin biopsy revealed perivascular IgM deposition and luminal blood fibrin thrombi in upper dermal capillaries, consistent with livedoid vasculopathy. Peripheral blood film showed red cell agglutination. Bone marrow biopsy identified a clonal CD5- CD10-small kappa-expressing B-cell population. A low concentration of monoclonal IgM-k paraprotein (3.9 g/L) was present (Table 1). Peripheral blood MYD88 mutation analysis was negative. Computed tomography (CT) scan of the neck, thorax, abdomen and pelvis did not reveal any organomegaly or lymphadenopathy. Apart from the livedoid rash and haematological parameters, he had no other clinical manifestations of lymphoma. As such, he was diagnosed with CBL-MZ; the livedo reticularis was attributed to IgM-K-mediated red cell agglutination. Treatment was not required as per prevailing guidelines [2]. During follow-up, he reported episodic visual blurring in either eye; aspirin was prescribed for possible ischaemic symptoms from increased plasma viscosity.

**Figure 2:**
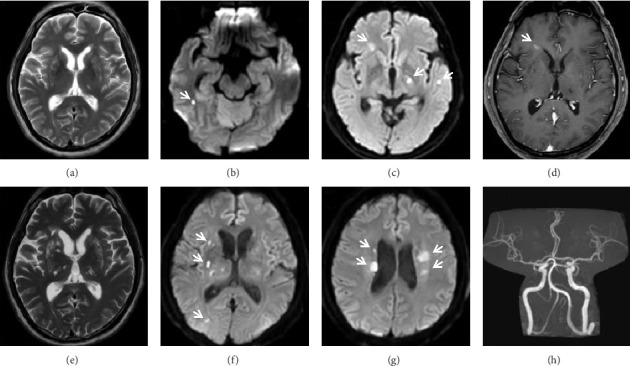
(a): Axial T2-weighted image performed during the patient's initial admission showing bilateral chronic subcortical infarcts. (b, c): Axial diffusion-weighted images during the patient's second admission showing small scattered acute and subacute subcortical infarcts. (d): Axial T1 postcontrast image demonstrating expected enhancement of the subacute infarct in the right subcortical frontal lobe, but the absence of other parenchymal, dural or leptomeningeal enhancement. (e): Axial T2-weighted image performed in subsequent admissions for stroke, demonstrating progressive subcortical white matter disease. (f, g): Axial diffusion-weighted images performed in subsequent admissions for stroke, showing further development of acute subcortical infarcts. (h): Normal intracranial magnetic resonance angiography.

Nine months later, he was hospitalised for recurrent transient visual blurring and unsteady gait. He described daily attacks of spontaneous nonpositional nonvertiginous giddiness, often associated with darkening of vision in the either eye, lasting up to 1 minute. Neurological and detailed ocular examinations were normal. Magnetic resonance imaging (MRI) of the brain only showed chronic subcortical infarcts (Figure 2(a)). His symptoms were attributed to increased plasma viscosity from underlying CBL-MZ. Aspirin was continued for secondary prevention of stroke.

**Figure 3:**
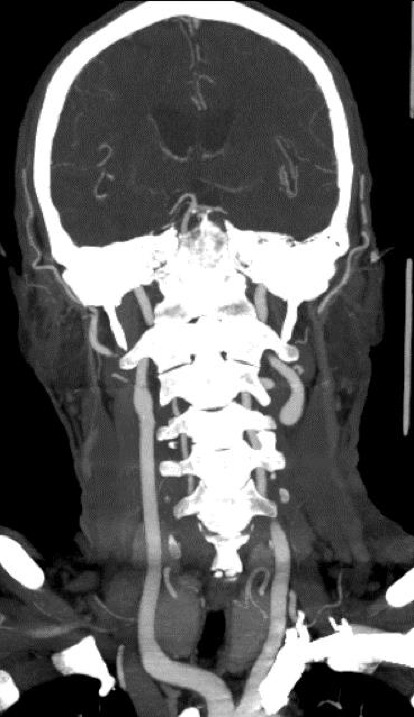
Coronal computed tomography angiography demonstrating normal extracranial carotid arteries.

Sixteen months following the diagnosis of CBL-MZ, he was again hospitalised for recurrence of episodic gait unsteadiness and visual blurring. Upon further history, he denied the presence of fever, B-symptoms, headaches, cognitive decline, illicit drug use or family history of cerebrovascular disease. The physical examination was again normal. Laboratory investigations showed normal lipid and glucose profiles. There was no significant change in his haematological profile compared to previous baseline (Table 1). Contrast-enhanced MRI of the brain revealed multiple small acute and subacute subcortical infarcts suggestive of a small vessel disease process (Figures 2(b), 2(c), 2(d)). Magnetic resonance angiography (Figure 2(h)) as well as CT angiogram of the intracranial and extracranial vessels did not show large vessel stenosis or occlusion (Figure 3). Extensive evaluation for secondary causes of stroke, in particular, tests for thrombophilia such as antiphospholipid syndrome, protein C/S, antithrombin III deficiency and prothrombin 20210G > A genotyping, as well as screens for systemic vasculitis, was unyielding (Table 1). Extended cardiac rhythm monitoring did not find atrial fibrillation; a transthoracic echocardiogram was normal. Transoesophageal echocardiogram (TOE) revealed a 7 mm filamentous mass on the aortic valve, likely a sterile vegetation. A tiny patent foramen ovale without right-to-left shunting was also visualised. Transcranial Doppler ultrasonography of both middle cerebral arteries (MCAs) did not show microembolic signals. Cerebrospinal fluid (CSF) analysis showed mild lymphocytic pleocytosis (19 WBC/uL) and elevated protein. Microbiology, including tests for herpes simplex virus-1 and -2 polymerase chain reaction (HSV-1/2 PCR), Gram stain and culture, fungal stains and culture, acid-fast bacilli smear, PCR and culture were negative. Oligoclonal bands were absent in both CSF and serum. Cytology, flow cytometry and MYD88 mutation analyses were also negative in the CSF. His neurological symptoms were attributed to stroke, for which the mechanism was thought to be cryptogenic at the time. Secondary prevention with antiplatelets and statins was started. Although nonbacterial thrombotic endocarditis remained a possibility, this invariably results in much larger multifocal infarcts.

**Figure 4:**
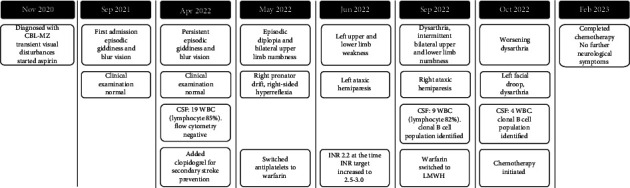
Timeline of events. Abbreviations: CBL-MZ, clonal B-cell lymphomatosis of marginal zone origin; CSF, cerebrospinal fluid; INR, international normalised ratio; LMWH, low-molecular-weight heparin; WBCs, white blood cells.

Despite optimal medical therapy for stroke prevention, including the combined use of aspirin and clopidogrel, and subsequently warfarin, the patient developed four further clinical strokes over the next 6 months (Figure 4). Brain MRI repeatedly demonstrated new multifocal deep and subcortical acute infarcts (Figures 2(e), 2(f), and 2(g)), consistent with a small vessel vasculopathy. His haematological profile remained unchanged. The CSF analysis was repeated twice, with both studies showing persistent mild lymphocytic pleocytosis, and both CSF flow cytometry now identifying a kappa-expressing clonal B-cell population with a similar immunophenotype to that found in the bone marrow 2 years earlier. The presence of a small vessel vasculopathy in the skin and CNS, CSF lymphocytic pleocytosis, raised inflammatory marker (ESR), and the exclusion of other aetiologies supported the diagnosis of a cerebral vasculopathy from lymphomatous meningitis leading to his recurrent ischaemic strokes. As CNS involvement of lymphoma was confirmed on flow cytometry of repeated CSF samplings, treatment was thus started. Had CSF sampling continued to be repeatedly unyielding, a brain biopsy would have been required. The patient received six cycles of rituximab, cyclophosphamide, vincristine, prednisolone and high-dose intravenous methotrexate. Following initiation of chemotherapy, he has remarkably had no further recurrence of ischaemic strokes in the past 2 years. Repeat CSF analysis after treatment showed normalisation of cell count and disappearance of lymphomatous cells.

## 3. Discussion

The evaluation of a patient with recurrent ischaemic stroke requires relevant investigations to determine the aetiology and hence initiate appropriate treatment. The mechanisms of ischaemic stroke can be divided into small vessel disease, large vessel atherosclerosis, cardioembolism and undetermined or other causes [3]. Imaging appearance of infarcts and vasculature are key in differentiating between the various mechanisms [4]. In this patient, the deep subcortical distribution of small infarcts in the territory of perforating arteries suggested a small vessel cerebral arteriopathy [5]. Although a potential cardioembolic source, the sterile vegetation seen on TOE was unlikely to have caused this patient's strokes as cardiac embolism tends to result in larger territorial or cortical infarcts, no microembolic signals were demonstrated on monitoring of the MCAs, and no benefit was seen despite therapeutic anticoagulation. A hypercoagulable state was also considered, but evaluation for systemic causes of prothrombosis was negative, d-dimer was not significantly elevated (Table 1), and infarcts in malignancy-related prothrombosis usually appear embolic in multiple territories, including the cortices [6], instead of the deep subcortical and basal ganglia location seen in our patient.

The differential diagnoses of a small vessel cerebral arteriopathy include inflammatory, infectious, iatrogenic, genetic, malignancy and paraneoplastic causes [7, 8]. Typical arteriosclerosis secondary to lipohyalinosis from chronic hypertension results in single infarcts in the distribution of penetrating cerebral arteries, unlike our patient who had recurrent multifocal infarcts despite optimisation of cardiovascular risk factors. Infectious and iatrogenic aetiologies were excluded from CSF microbiology and the absence of previous exposure to inciting agents respectively. He had no family history of cerebrovascular disease, and the rapid clinical progression made a genetic cause unlikely. There were no features of systemic autoimmunity, and antibodies for systemic vasculitis were absent. The combination of recurrent strokes and livedoid rash raised the possibility of Sneddon syndrome, which is a noninflammatory small- and medium-sized vasculopathy of the CNS, skin and, in some cases, the cardiac valves and kidneys [9]. However, unlike our patient, the CSF in Sneddon syndrome is usually normal, and there tends to be a response to anticoagulation. Intravascular lymphoma, an aggressive large B-cell non-Hodgkin lymphoma arising within the lumen of small blood vessels, can also appear as infarcts [10], but the lesions in intravascular lymphoma are typically cortical, and there was no evidence of aggressive transformation of the clonal B cells found on repeated CSF samplings. Primary (or granulomatous) angiitis of the CNS (PACNS) may present with multifocal ischaemic strokes and CSF pleocytosis. Indeed, PACNS has previously been described in association with Hodgkin's disease [11]. Although our patient's imaging showed developing leukoencephalopathy consistent with small vessel vasculitis, PACNS was excluded as clonal B-cells were found in the CSF.

In patients with haematological disorders and CNS manifestations, other differentials including hyperviscosity syndrome, cryoglobulinemic vasculitis, Bing–Neel syndrome, and CNS involvement of multiple myeloma ought to be considered. In hyperviscosity syndrome, mucosal bleeding, retinal hemorrhages or thrombosis, cerebral haemorrhage or seizures can occur; ischaemic strokes are rare, as viscosity is highest in small venules. The diagnosis is made with significantly elevated monoclonal paraprotein concentrations or serum viscosity, not seen in our patient [12]. Cryoglobulinemic vasculitis can rarely cause a cerebral vasculitis manifesting as recurrent ischaemic strokes [13] and is confirmed by the presence of cryoglobulins in the serum, which was repeatedly negative in our patient. Bing–Neel syndrome results from CNS infiltration by lymphoplasmacytic cells from underlying Waldenstrom macroglobulinemia [14], while multiple myeloma can rarely involve the CNS [15]. Waldenstrom macroglobulinemia, MZL and multiple myeloma can all lead to monoclonal IgM in the peripheral blood. These diagnoses can be differentiated based on the bone marrow analysis and immunophenotyping of the neoplastic cells. In Bing–Neel syndrome and CNS involvement of multiple myeloma, rather than infarcts, brain MRI may show contrast enhancement and/or thickening of the meninges, or focal contrast-enhancing lesions in the cerebral parenchyma, which were not observed in our patient.

Cerebral vasculopathy has been reported in the context of Hodgkin lymphoma as well as other solid-organ tumours [8, 11, 16] but has never previously been associated with CBL-MZ or MZL. Mixed cryoglobulinemia can be a paraneoplastic phenomenon of MZL [17] and has been reported to cause a systemic vasculitis without cerebral involvement resulting from mixed cryoglobulinemia [18]. An infectious cerebral vasculopathy secondary to HSV-1 in the context of MZL has also been described [19]. A patient with gastric mucosa-associated lymphoid tissue (MALT) lymphoma was reported to develop multifocal cerebral infarctions, although neoplastic involvement of the CNS was not proven [20]. To the best of our knowledge, cerebral vasculopathy from MZL leading to ischaemic stroke has not been reported in the literature. Postulated mechanisms for neoplasms leading to cerebral vasculopathy include cytokine-induced endothelial injury, an immunologic reaction affecting the vascular endothelium, or direct effects of tumoral cells on vessel walls [21]. We hypothesise that the presence of clonal B-cells in our patient's CSF may have led to a small vessel vasculopathy either through a paraneoplastic process or from direct effect of the lymphoma.

CNS involvement in MZL usually occurs as dural-based lesions [22], although there are rare cases of diffuse leptomeningeal involvement presenting with visual disturbances and papilloedema [23, 24]. Our patient had stable haematological parameters without evidence of progression to overt MZL, and such did not meet conventional criteria for treatment initiation. The lack of clear precedence in the literature led to a diagnostic and therapeutic dilemma. To avoid further neurological disability, multidisciplinary discussions concluded that treatment of the underlying CBL-MZ, the most likely cause of his recurrent strokes, should be undertaken. The immediate cessation of further episodes of stroke following chemotherapy supported this decision and attributed neoplastic meningitis from CNS involvement of CBL-MZ as the causative factor.

## 4. Conclusion

This case highlights that even indolent lymphomas can result in clinically significant CNS spread, which should prompt clinicians to consider treatment. We also briefly discuss the approach to recurrent stroke, the importance of pattern recognition on imaging to differentiate between the various mechanisms of ischaemic stroke, and the approach to small vessel cerebral vasculopathy.

## Figures and Tables

**Table 1:** Laboratory results performed at the diagnosis of CBL-MZ and at the presentation for ischaemic stroke.

	Reference range	Result	
		At diagnosis of CBL-MZ 16 months prior to current admission	At initial presentation for ischaemic stroke
WBC (×10^9^/L)	4.0–9.6	19.5	16.9
Neutrophils	1.90–6.60	7.80	3.70
Lymphocytes	1.10–3.10	9.97	11.99
Monocytes	0.20–0.70	1.20	0.77
Eosinophils	0.00–0.60	0.41	0.34
Haemoglobin (g/dL)	13.6–16.6	14.6	13.8
Platelets (×10^9^/L)	150–360	394	330
Prothrombin time	27.0–37.0	32.6	33.8
Activated partial thromboplastin time	11.7–14.0	13.2	13.1
LDH (U/L)	270–550	329	263
Haptoglobin (mg/dL)	36–200	261	174
Direct coombs, polys		Not sent	Positive (1+)
Direct coombs, IgG		Not sent	Negative
DCT anti-C3b, C3d		Not sent	Positive (weak +)
IgM (d/L)	0.4–2.4	3.9	4.0
D-dimer (*μ*g/mL)	< 0.50	Not sent	1.24
ESR (mm/hr)	1–10	Not sent	65
Lupus anticoagulant		Absent	Absent
Anti-B2-glycoprotein (RU/mL)	0–20	Not sent	< 2
Anticardiolipin IgM (MPL units)	0–20	Not sent	< 20
Anticardiolipin IgM (GPL units)	0–20	Not sent	< 20
APC resistance test	0.9–1.3	Not sent	0.9
Anti-thrombin III (%)	80–130	Not sent	93
Protein S activity (%)	65–130	Not sent	73
Protein C activity (%)	70–150	Not sent	85
Prothrombin 20210G > A genotyping	Normal	Not sent	Normal
ANA (titre)	< 80	< 80	< 80
Anti-ds-DNA (IU/mL)	0–25	Not sent	< 25
ANCA	Negative	Not sent	Negative
Homocysteine (*μ*mol/L)	5–15	Not sent	13
Syphilis IgG ELISA (RU/mL)	< 16	Not sent	4
HIV screen		Not sent	Non-reactive

Abbreviations: ANA, antinuclear antibody; ANCA, antineutrophil cytoplasmic antibody; anti-ds-DNA, antidouble stranded deoxyribonucleic acid; APC, activated protein C; ESR, erythrocyte sedimentation rate; HIV, human immunodeficiency virus; LDH, lactate dehydrogenase; MZL, marginal zone lymphoma; WBCs, white blood cells.

## Data Availability

The data that support the findings of this study are available from the corresponding author upon reasonable request.
